# *Trans* isomeric fatty acids in human milk and their role in infant health and development

**DOI:** 10.3389/fnut.2024.1379772

**Published:** 2024-03-07

**Authors:** Okba Hatem, Ömer Furkan Kaçar, Hüsna Kaya Kaçar, József L. Szentpéteri, Tamás Marosvölgyi, Éva Szabó

**Affiliations:** ^1^Doctoral School of Health Sciences, Faculty of Health Sciences, University of Pécs, Pécs, Hungary; ^2^Department of Biochemistry and Medical Chemistry, Medical School, University of Pécs, Pécs, Hungary; ^3^Department of Nutrition and Dietetics, Faculty of Health Sciences, Amasya University, Amasya, Türkiye; ^4^Institute of Transdisciplinary Discoveries, Medical School, University of Pécs, Pécs, Hungary; ^5^Institute of Bioanalysis, Medical School, University of Pécs, Pécs, Hungary

**Keywords:** development, human milk, infant, newborn, nutrition, review, *trans* fatty acid

## Abstract

It is well known that long chain polyunsaturated fatty acids (LCPUFAs) play an important role in neurodevelopment in the perinatal life. The most important source of these fatty acids is the diet, however, they can also be formed in the human body from their shorter chain precursors, the essential fatty acids. Since the WHO recommends exclusive breastfeeding for the first six months after birth, the exclusive source of these fatty acids for breastfed infants is human milk, which can be influenced by the mother’s diet. Unsaturated fatty acids can have either cis or *trans* configuration double bond in their chain with distinct physiological effects. Cis isomeric unsaturated fatty acids have several beneficial effects, while *trans* isomers are mostly detrimental, because of their similar structure to saturated fatty acids. *Trans* fatty acids (TFAs) can be further subdivided into industrial (iTFA) and ruminant-derived *trans* fatty acids (rTFA). However, the physiological effects of these two TFA subgroups may differ. In adults, dietary intake of iTFA has been linked to atherosclerosis, insulin resistance, obesity, chronic inflammation, and increased development of certain cancers, among other diseases. However, iTFAs can have a negative impact on health not only in adulthood but in childhood too. Results from previous studies have shown that iTFAs have a significant negative effect on LCPUFA levels in the blood of newborns and infants. In addition, iTFAs can affect the growth and development of infants, and animal studies suggest that they might even have lasting negative effects later in life. Since the only source of TFAs in the human body is the diet, the TFA content of breast milk may determine the TFA supply of breastfed infants and thus affect the levels of LCPUFAs important for neurodevelopment and the health of infants. In this review, we aim to provide an overview of the TFA content in human milk available in the literature and their potential effects on infant health and development.

## 1 Introduction

The Food and Drug Administration (FDA) provides a crucial definition of *trans* fatty acids (TFA) as “the sum of all unsaturated fatty acids that contain one or more isolated (i.e., non-conjugated) double bonds in a *trans* configuration” (1). Almost all naturally occurring unsaturated fatty acids have double bonds in a cis configuration, while natural TFAs are formed in the rumen of ruminants (rTFA). Other types of *trans* isomers are formed during industrial processes, such as the partial hydrogenation of vegetable oils or the heating of oils at high temperatures (iTFA). Consequently, the primary sources of TFAs in human diets encompass milk and meat from ruminant animals, products containing partially hydrogenated oils (PHOs), and various foods subjected to cooking methods such as deep-frying and baking (2).

Research over the last 30 years has highlighted the negative effects of TFAs, including the direct link between increased dietary TFA intake and a worrying increase in cardiovascular disease (CVD) (3). TFA consumption may also reduce insulin sensitivity, increase diabetes risk, can act proinflammatory, impair endothelial function (4), and also can be associated with an increased risk of breast cancer (5). These detrimental effects were mainly attributed to the widespread use of PHOs, which serve as a fundamental source of TFAs, specifically iTFAs (6). As a result of the detrimental effects of iTFAs, the World Health Organization (WHO) announced an action plan called REPLACE, which aims to eliminate iTFA from the global food supply by the end of 2023 (7). However, of more important significance for the development of breastfed infants is the fact that several studies have suggested that *trans* isomers may interfere with the metabolism of long-chain polyunsaturated fatty acids (LCPUFAs), which are important for neurodevelopment (8–10). Since TFAs cannot be synthesized in the human body, diet is the only way to obtain these fatty acids. Hence, for breastfed children, human milk (HM) is the only source of TFAs and their potential adverse effects (11).

Breastfeeding is the optimal nutritional method for infants, offering a plethora of health benefits, not only for the infant but also for the mother. This natural feeding approach promotes healthy development and long-term benefits for breastfed infants (12). Public health organizations, including the WHO and the American Academy of Pediatrics (AAP), recommend exclusive breastfeeding for infants up to the age of 6 months (13–15). Consequently, HM is the only source of nutrients for breastfed infants during their first months of life to support their growth and development. However, the composition of HM is not constant, but it adapts to the different needs of the infant throughout lactation, including colostrum (C), transitional milk (TM), and mature milk (MM) (16). Moreover, it can differ depending on demographics (17) and genetic factors (18), as well as maternal diet and lifestyle (19). Maternal dietary changes seem to have a greater impact on the fat content of HM than on its protein and carbohydrate levels (20). The fatty acid (FA) composition of HM reflects the maternal dietary fat alterations over a period of 1–3 days (21).

In the perinatal period, LCPUFAs, mainly arachidonic acid (C20:4n-6, AA) and docosahexaenoic acid (C22:6n-3, DHA), play an important role in the neurodevelopment and visual acuity (22), because they serve as important components of the membranes of the retinal photoreceptors and brain cells (23). The only sources of these FAs during this period are maternal stores and the mother’s diet. During the intrauterine period, these FAs are transferred to the fetus via the placenta and after birth through HM. Previous animal studies suggest that TFAs may decrease the activity of hepatic delta-6 desaturase, thereby interfering with the metabolism of physiologically important LCPUFAs (24, 25). The human body is not able to synthesize TFAs, so these fatty acids are ingested through our diet. As the disturbed metabolism of LCPUFAs by TFAs in early life may have a longer-term effect, we aim to provide an overview of the TFA content of HM worldwide based on previous publications and to summarize the results of animal models and human studies on its potential effects on infants and children.

## 2 Fatty acids in general

### 2.1 Nomenclature and metabolism

**Figure 1:**
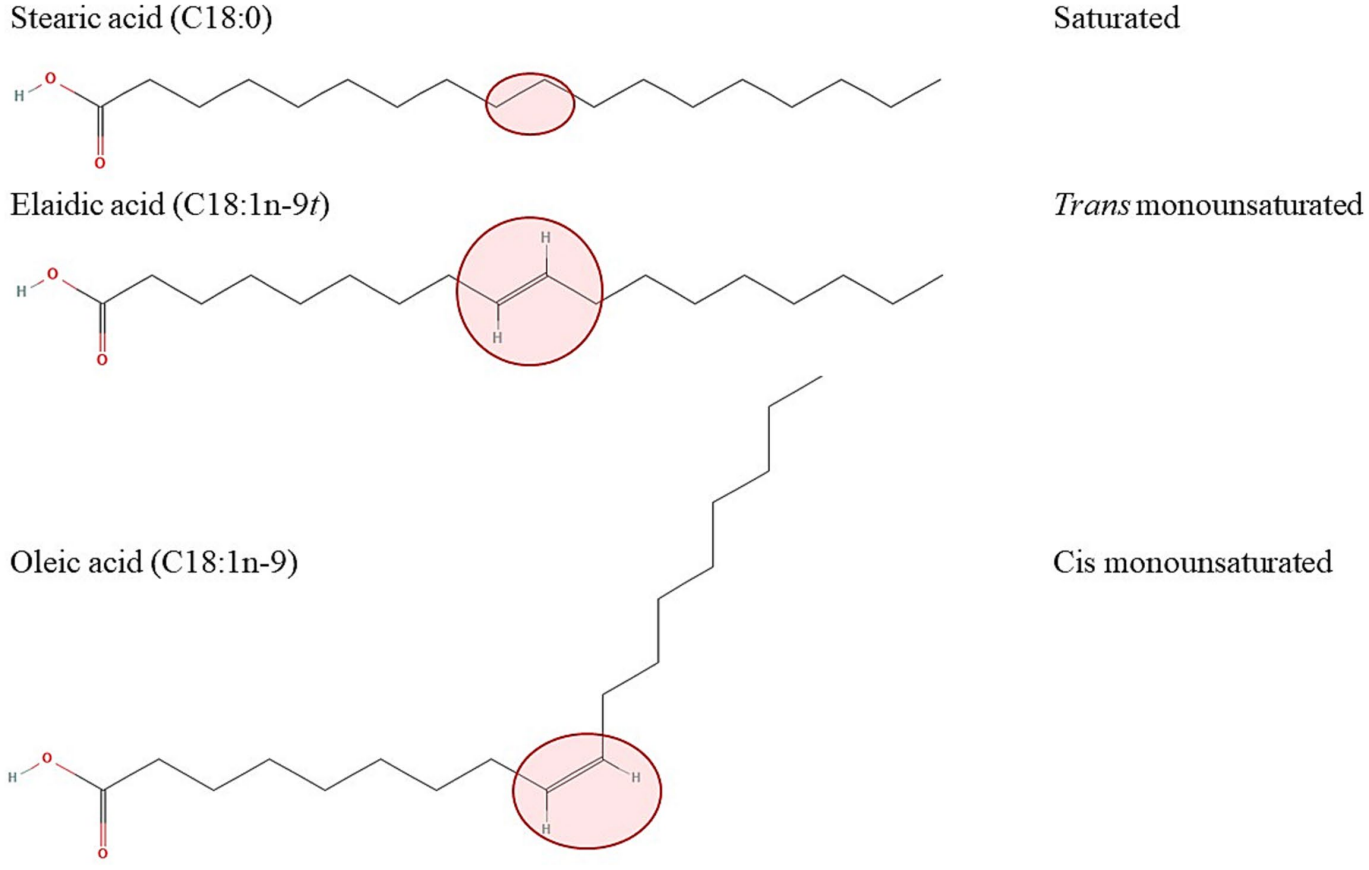
Effect of a configuration of the double bond on the spatial arrangement of the molecule with the example of 18-carbon saturated (stearic acid, top) *trans* (elaidic acid, middle) and cis (oleic acid, bottom) monounsaturated fatty acids. The structures of the fatty acids were downloaded from: https://pubchem.ncbi.nlm.nih.gov/compound/5281#section=2D-Structure (stearic acid), https://pubchem.ncbi.nlm.nih.gov/compound/637517#section=2D-Structure (elaidic acid), https://pubchem.ncbi.nlm.nih.gov/compound/oleic%20acid#section=2D-Structure (oleic acid).

FAs are carboxylic acids with a carboxyl group at one (alpha end) and a methyl group (omega end) at the opposite end. Almost all naturally occurring FAs hold the double bonds in the cis configuration, where the hydrogen atoms on the double bond are on the same side of the molecule, causing a bend in the chain, while in *trans* isomers the hydrogen atoms are on opposite sides of the molecule. This results in a more stable and rigid chain, making it comparable to saturated fatty acids (Figure 1) (3, 26).

TFAs can be formed through industrial processes or naturally. iTFAs are created during food preparation at high temperatures or through partial hydrogenation of vegetable oils, which involves high temperatures, high pressures, and a catalyst. In this process, polyunsaturated fatty acids (PUFAs) undergo partial saturation of their double bonds to decrease melting point. Additionally, some double bonds in cis configuration are converted to a *trans* configuration. Several different mono-and polyunsaturated *trans* isomers can be formed as a result, with the double bond positioned at delta-6 or higher. However, the most prominent ones are the monounsaturated *trans* isomers of C18. Elaidic acid (EA, C18:1n-9 *t*), which is the geometrical isomer of oleic acid (C18:1n-9), has the highest prevalence in iTFAs. The most prevalent polyunsaturated TFA is linoelaidic acid (C18:2n-6*tt*), which is the *trans* isomer of the essential n-6 linoleic acid (LA, C18:2n-6) and the *trans* double bonds are in the delta-9 and delta-12 positions (3, 27).

In contrast, rTFAs are formed in the rumen of ruminants through microbial fermentation and biohydrogenation. Although the *trans* double bonds in rTFAs may be in a similar position to those in iTFAs, the probability of formation and frequency of occurrence differ. The most prominent rTFA is *trans* vaccenic acid (VA, C18:1n-7), which has a double bond in the delta-11 position. Moreover, ruminants also produce a group of conjugated linoleic acid (CLA), in which the double bonds are conjugated. This means that there is only one single bond between the two double bonds. The most prevalent CLA is rumenic acid (RA, C18:2n-9c7*t*) (28). Not only the position of the *trans* double bond can differ between the iTFAs and rTFAs, but also their concentration in foods. The concentration of iTFAs in products can reach up to 50% of the total fatty acids, whereas rTFAs in ruminant milk are typically found in much lower concentrations, usually around 4–6% of the total fatty acids. The most common TFA isomers are listed in Table 1 with their names and origin.

**Table 1:** Most common *trans* isomers with name, origin, and formula.

Origin of TFA	Name	Structure	Systematic name
rTFA	Palmitelaidic acid	C16:1n-7 *t*	9E C16:1
rTFA	*trans* Vaccenic acid	C18:1n-7 *t*	11E C18:1
iTFA	Elaidic acid	C18:1n-9 *t*	9E C18:1
iTFA	Linoelaidic acid	C18:2n-6*tt*	9E,12E C18:2
rTFA	Ricinenic acid	C18:2n-9 *t*,7 *t*	9E,11E C18:2
rTFA	Rumenic acid	C18:2n-9c,7 *t*	9Z,11E C18:2

iTFA, industrial *trans* fatty acid; rTFA, ruminant *trans* fatty acid; TFA, *trans* fatty acid.

Dietary TFAs can be absorbed from the small bowel, incorporated into serum triacylglycerols (TGs) and structural lipids, and stored in adipose tissue (29, 30). However, TFAs are preferentially incorporated into TGs and phospholipids (PLs), and to a lesser extent into cholesterol esters (31). There may also be differences in tissue incorporation, for example, delta-9 and delta-11 C18:1 *t* TFAs are incorporated into tissues to a greater extent than other positional *trans* isomers (32). However, this incorporation is not irreversible, but rather dynamic, so that when dietary intake of TFAs decreases, they are released from membranes. The *trans* double bond is recognized by enzymes in a similar way to the saturated bond, so EA can be incorporated into phospholipids at the expense of saturated fatty acids (e.g., stearic acid), or cis-9, cis-12, *trans*-15 C18:3 can be incorporated into cardiolipins instead of LA (33). The effect of TFAs in membranes is similar to that of saturated FAs, because they both have a more rigid structure than cis unsaturated fatty acids, so they both decrease membrane fluidity and therefore the affinity of molecules for receptors.

**Figure 2:**
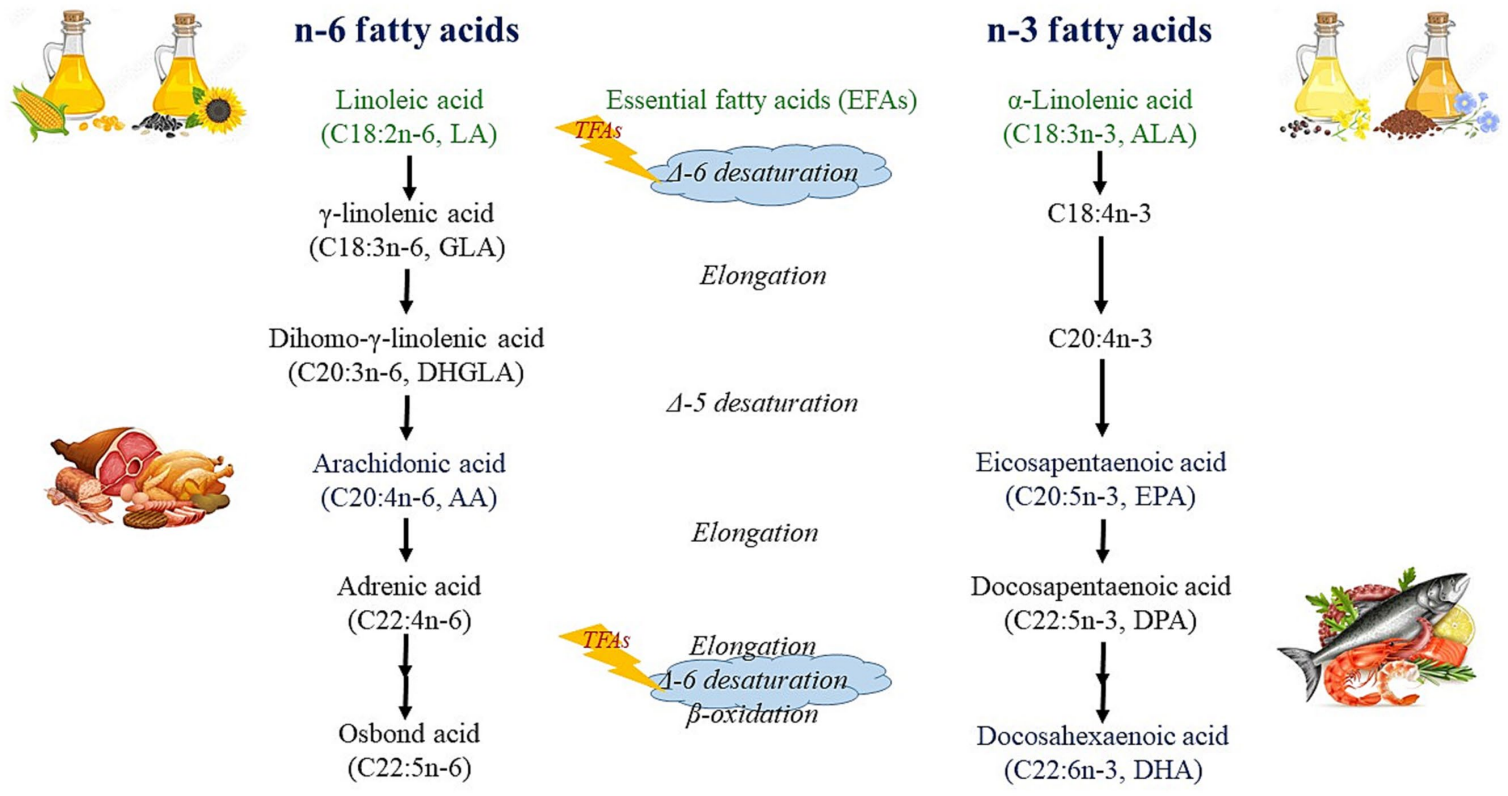
Metabolism of n-6 and n-3 fatty acids.

TFAs have been associated with a number of adult diseases and consequent death cases, such as atherosclerosis, cardiovascular disease and mortality (34, 35), and cancer (36). Animal studies have indicated that TFAs may inhibit the activity of the Δ-6 desaturase enzyme, disrupting the production of omega-3 and omega-6 LCPUFAs (Figure 2) (37). This interference may have significant effects on newborns as the activity of these enzymes is required for the synthesis of LCPUFAs (38, 39). As a matter of fact, the presence of TFAs in milk may cause an insufficient supply of essential fatty acids (EFAs) in both HM (38, 40) and infant blood (39). Some TFAs can also be metabolized in the body, for example, the human body can synthesize RA from VA in healthy adults (41), and also in lactating women (42).

The *de novo* synthesis of LCPUFAs is rather limited and therefore, infant levels of these FAs depend primarily on maternal dietary intake (43). In contrast, TFAs cannot be synthesized within the body and are entirely dependent on maternal diet. Therefore, a high concentration of TFAs could potentially have a negative impact on the supply of LCPUFAs due to direct competition with metabolic enzymes. It is important to note that there is a positive correlation between the concentrations of most FAs, including TFAs, EFAs, and LCPUFAs, in HM, and their corresponding levels in the infant’s plasma, highlighting the significance of HM in the dietary intake of these FAs (9). On the other hand, the significant inverse relationship between the concentrations of TFAs and EFAs (LA and α-linolenic acid [C18:3n-3, ALA]) in both preterm (44) and term HM (9, 45) suggests a restrictive effect of TFAs on the EFA supply. These results support the need to ensure adequate EFA supply to infants by increasing EFA intake in the maternal diet on the one hand and by limiting TFA consumption on the other.

### 2.2 Fatty acids in the diet

HM is the best option for infants in the first months of life (46) and therefore exclusive breastfeeding is recommended until the 6th month of life (15). Fat in HM is the primary source of energy (47), while selected FAs, as well as crucial fat-soluble vitamins are essential for the development of infants (48, 49). The FAs of HM are either synthesized in the mammary gland (MG) or derived from plasma FAs that originate from lipid reserves, non-mammary tissues, or diet (47). Depending on the mother’s intake, the concentration of FAs in HM is influenced by the type of diet prevalent in the area where the mother lives (11, 50–53). Therefore, a well-balanced diet is beneficial to both the mother and the breastfed child throughout the period of breastfeeding (54).

Maternal reserve capacity and the metabolic utilization of FAs (transport, synthesis, and oxidation) are directly related to FA levels during lactation (55). It has been estimated that between 60 and 70% of the fat in HM is derived from tissue synthesis and maternal fat deposits, while approximately 30% is derived from the diet of the mother (40, 50). This suggests that maternal diet, as well as the metabolism of FAs during lactation appear to be the most important factors that influence DHA concentration in HM (56). Additionally, the composition of fats in HM undergoes continuous change due to the rapid transport of dietary fats from chylomicrons. This process results in a peak occurring within 6 to 12 h after dietary DHA intake (55). Isotopic studies show, that about 30% of dietary LA intake is directly transferred to HM, but only a maximum 3% is converted and transferred as AA (57, 58). Although approximately 65% of HM ALA is derived from maternal dietary ALA intake, the low efficiency of conversion to its longer-chain metabolites and transfer to HM means that dietary ALA plays a negligible role in the eicosapentaenoic acid (C20:5n-3, EPA) and DHA content of HM (59).

TFAs cannot be synthesized *de novo* by the human body (60), so their exclusive sources are dietary intake. Ruminant animals naturally produce rTFAs through a process known as biohydrogenation in the rumen. Therefore, meat, milk and dairy products from ruminant animals (cattle, sheep, and goats) are the primary sources of rTFAs in the human diet. Conversely, iTFAs are primarily formed during the hydrogenation of vegetable oils during industrial processes of hardening and deodorization, so iTFAs are primarily consumed through PHOs and food products that contain them, including confectionery and food concentrates, but a small amount of iTFAs are also produced during the thermal processing of food, such as baking or frying (61, 62). The main iTFA present in the food supply is EA (27), which has been detected in larger proportions in HM (38, 40, 63, 64), but other iTFAs, like linoelaidic acid, have also been reported (40, 50, 63, 64). The most prevalent rTFA, found in milk, butter, and beef fat, is VA (26, 27), which has also been detected in HM by several research groups (40).

Based on reported data worldwide, rTFA intake from ruminant products, including meat, milk, and butter, constitutes a small, but constant portion of total TFAs, while the percentage of iTFAs derived from processed foods, bakeries, snacks, and fast food is continuously declining in most countries (65, 66). Bakery products, confectionery, and snack foods accounted for the majority of TFAs in the diets of breastfeeding women on a typical Western diet (11, 67) as well as on a Mediterranean diet (38). Moreover, maternal diet influences the HM TFA concentration with a significantly lower value (0.44%) in vegan compared to vegetarian (0.66%) and omnivorous (1.09%) lactating women (52). Previous studies have analyzed the fatty acid composition of various food categories containing TFAs. In general, older studies reported higher iTFA values, resulting in higher total TFA content in these foods. In a study conducted in Sweden (68), the total TFA content in certain foods was as high as 3.83 w/w% (digestive biscuits) during the period of 1995–1997. In contrast, Germany had even higher total TFA values in 2007–2009 (27): the highest levels were found in certain chocolate products (0.05–40.46 w/w%), doughnuts (0.14–35.11 w/w%), French fries and chips (0.12–16.30 w/w%), and biscuits (0.04–10.66 w/w%).

Considering the detrimental impact of iTFAs on human health, the European Food Safety Authority (EFSA) advises that TFA intake should be restricted to the lowest achievable level in a nutritionally balanced diet (69, 70). Accordingly, the World Health Organization (WHO) recommends limiting consumption of iTFA to less than 1% of daily energy intake to prevent chronic diseases, due to scientific evidence demonstrating its harmful effects (71, 72). Several studies have also examined the impact of regulatory or voluntary restrictions on the total TFA content in various food groups. In Spain, certain products, such as chocolate-stuffed Swiss rolls (1.47–11.85 w/w%), doughnuts (8.47–10.89 w/w%), and chocolate-filled sponge cake (0.6–9.12 w/w%), had high TFA content due to their high iTFA content in 1999 (73). However, the values greatly decreased after regulation by 2010 and further decreased by 2015 (74), e.g., in confectionary and pastry products (2010: 0.657 w/w%; 2015: 0.034 w/w%), chocolate products (2010: 0.721 w/w%; 2015: 0.14 w/w%), and biscuits (2010: 0.311 w/w%; 2015: 0.008 w/w%). By contrast, the TFA content of mainly rTFA-containing products, such as dairy products, remained quite stable. For example, butter had a TFA content of 2.356 w/w% in 2010 and 2.452 w/w% in 2015, while spreadable cheese contained 2.646 w/w% and 2.524 w/w% TFA, respectively. Similar trends were observed in Sweden with the highest total TFA values in 1995–1997 and really low values in 2007 (68) as well as in Argentina (75) and Korea (76). Table 2 summarizes the total TFA values of various food groups based on recent measurements in Thailand (77), Egypt (78), Argentina (75), Spain (74), Jamaica (79) and Malaysia (80). Previous research has shown that both voluntary and regulatory measures to reduce iTFA content in foods have resulted in substantial reductions in global intakes of TFAs over the past two decades (61, 81, 82), including Canada (83) and the European Union (EU) countries (84). The estimated intake of TFAs in 2010 varied widely from 0.2 E% (Barbados) to 6.5 E% (Egypt) (82), while seven years later, intake was lower, ranging from 0.3 E% (China) to 4.2 E% (Iran). In addition, the average intake was below the maximum recommended intake of 1 E% in 22 out of the 29 countries (61). According to the most recent study, TFA intake was below 0.8 E% in most regions across the world, with the highest reported intake in North-, Central-, and Latin America, with levels ranging from 0.8 E% to 1.2 E% (85).

**Table 2:** Total TFA content (w/w%) of some food groups based on recent studies worldwide.

Food category	Country (year of TFA determination)					
	Thailand (2017) (77)	Egypt (2019) (78)	Argentina (2015) (75)	Spain (2015) (74)	Jamaica (2019) (79)	Malaysia (n.d.) (80)
rTFA-containing products						
Butter	2.04–4.64	1.2		2.452		0.11–3.85
Cheese	0.68–1.08	1.5				0.33–2.57
Milk	0.08–0.11	2.1				
Whole milk powder	1.32	1.4				
iTFA-containing products						
Biscuits		2.0		0.008		
Chocolate and cocoa products		2.6		0.14		
Crackers		1.3		0.011	0.91	
Potato chips		1.5	0.4	0.088	0.87	0.05–0.50
French fries		0.5				0.09–0.51
Margarine			1.1	0.274	0.09	0.05–0.54
Confectionary, doughnut, pastries			3.7	0.721	0.06	

n.d. denote no exact data is given; TFA, *trans* fatty acid.

## 3 *Trans* isomeric fatty acids in human milk

### 3.1 *Trans* fatty acid content of human milk worldwide and its influencing factors

Maternal diet-derived TFAs can be transferred directly to HM (86). Based on the dietary sources and intake of TFAs, HM contains varying levels of TFAs across different populations and regions of the world (87). This systematic literature review found the highest TFA values in North America (6.99 [5.01–8.98]; mean [95% confidence interval for mean]), followed by South America (2.36 [1.14–3.58]), Europe (1.82 [1.61–2.03]) and Oceania (1.21 [1.19–1.23]), and the lowest values in Asia (0.71 [0.65–0.78]) and Africa (0.61 [0.55–0.67]). However, it is also important to consider that the TFA of HM is also dependent on the time of analysis. Higher values were reported in the literature prior to the implementation of the iTFA regulation. For instance, a Canadian study (83) investigated the effect of regulation on the TFA content of HM samples and found that the high values reported before regulation (7.2 ± 0.3% in 1992) were significantly reduced (1.9 ± 0.5% in 2011).

**Figure 3:**
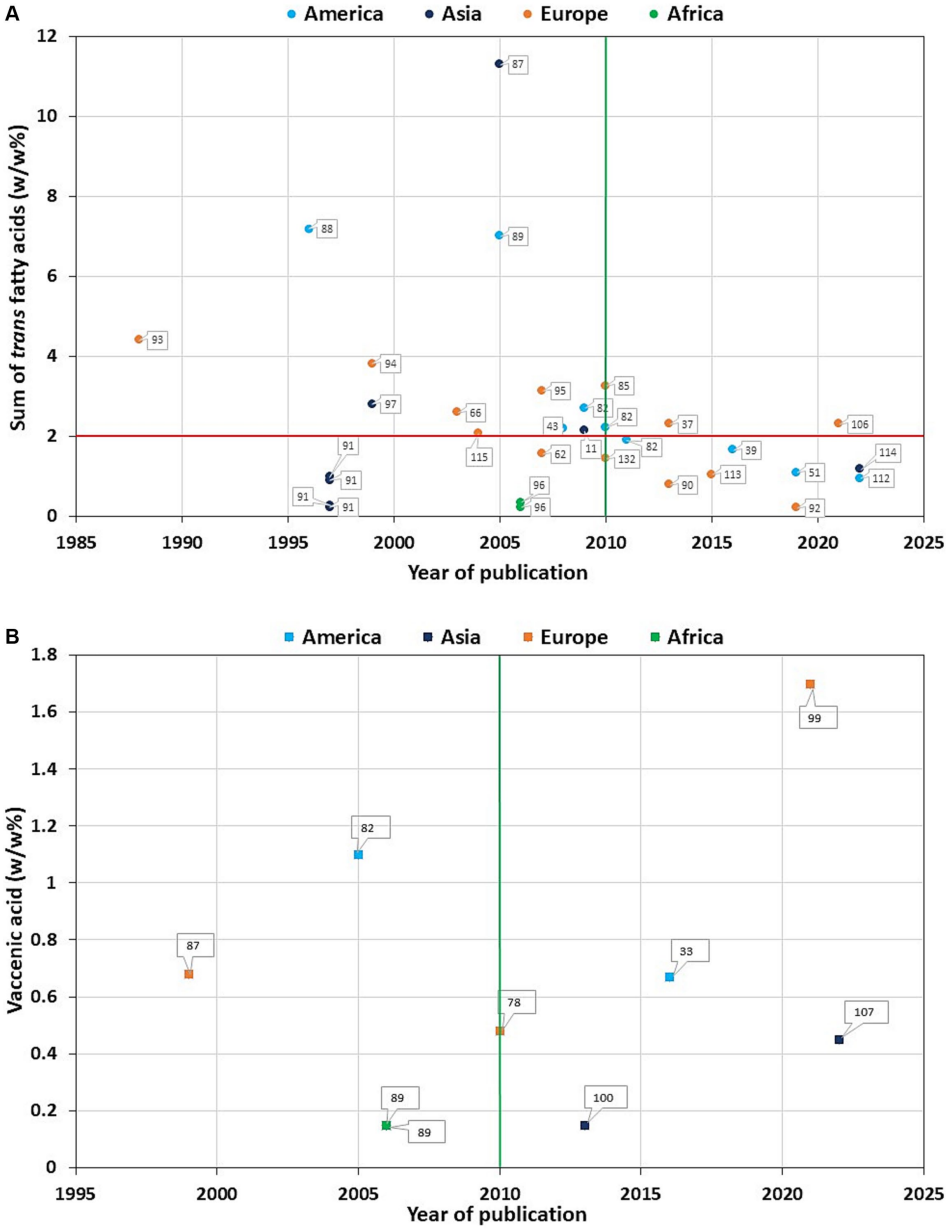
Total *trans* fatty acid **(A)** and *trans* vaccenic acid **(B)** values in mature human milk as a function of year of publication. The numbers assigned to each data item correspond to the article number in the bibliography. The vertical green line indicates the year 2010, the horizontal red line indicates the total *trans* fatty acid value of 2 w/w%.

In the literature we can find a wide variety of TFA levels in HM among different populations (Supplementary Tables S1, S2), which range from the highest level in Iranian (88), Canadian (45, 89), and American (90) to the lowest level in Greek (91), Chinese (92) and Latvian lactating mothers (93). However, when dividing the literature into two groups based on the time of the study, it becomes apparent that the studies reporting very high levels of total TFAs are older, probably prior to the TFA regulation (Figure 3A). Total TFA values of 3% or more are exclusively found in studies published in 2010 or before (86, 88–90, 94–96), while very low TFA values of less than 0.5% are found in articles published both before (92, 97) and after 2010 (52, 91, 93, 97). However, this 2010 cut-off date is somewhat arbitrary, as the TFA legislation was adopted at different times around the world. It is worth noting that prior to 2010, neither Iran nor Kuwait had regulations on the TFA content of food, although Kuwait (98) had significantly lower levels of TFA in HM compared to Iran (88). According to WHO, only seven countries had best-practice TFA policy before 2017, including Denmark (since 2004), Austria (since 2009), Chile, Iceland, South Africa (all three since 2011), Hungary and Norway (both since 2014). In 2018 this best-practice TFA policy was implemented in Canada, Latvia, Slovenia, and USA, in 2019 in Lithuania and Thailand, and 2020 in Saudi Arabia also (99). Considering the increasing number of countries applying the regulation, it is expected that the amount of TFAs in HM will decrease further. However, the intake of rTFA remains constant, resulting in no change in the VA content of HM between before and after 2010 (Figure 3B).

The FA composition of HM, including the TFA content, can be influenced by several factors. One of these may be ethnicity, which probably leads to differences in HM TFA content due to distinct dietary habits. For example, in a Czech study, Roma women had significantly higher mean C18:1 t levels in their HM than the average Czech population (2.73 ± 1.88 vs. 2.09 ± 1.24; w/w%, mean ± SD). The difference was explained by the higher consumption of foods with higher TFA content among Roma women, including buns, biscuits, butter, hamburgers, potato chips, and other fried food (96). This was also corroborated in an Asian study, where Chinese women on a Western-style diet had significantly higher TFA levels in their HM compared to lactating women on a traditional Chinese diet (92, 100). Similarly, different C18:1n-9 *t* values were found in an African country, Nigeria, when comparing the HM of urban and nomadic people (0.08 ± 0.07 vs. 0.03 ± 0.02, w/w%, mean ± SD), although no significant difference was found for most of the measured TFA values (97). However, if there is no difference in dietary habits, then ethnicity alone has no detectable effect on the amount of TFA in HM (63, 101). A Polish study found that EA values in HM was not significantly influenced by body fat, maternal BMI, health problems or n-3 LCPUFA supplementation (102).

When examining groups with differing ethnicities and geographic locations, even larger differences can occur. In HM samples from German mothers on a Western-style diet, significantly higher TFA levels were found in almost all measured TFA isomers compared to Nigerian mothers still on a traditional diet [C14:1 *t* 0.19 vs. 0.04, *p* = 0.0009; C16:1 *t*: 0.46 vs. 0.27, *p* = 0.001; C18:1 *t* 3.12 vs. 0.86, *p* = 0.0017; sum of TFAs: 4.40 vs. 1.20, *p* = 0.0012; w/w%, median (103)]. Similarly, large differences were measured in the TFA content of HM between US women on a Western-style diet (high in iTFAs) and Bolivian forager-horticulturalists (iTFA-free, but containing rTFAs); C18:1 *t* was about six times higher than in Bolivia [1.23 ± 1.05 vs. 0.21 ± 0.30; w/w%, mean ± SD (104)]. Although TFA regulation in recent years has resulted in much lower TFA intakes worldwide, it seems that Western-style diets still result in higher TFA intakes compared to traditional diets (sum of TFAs: Australia: 1.21 vs. Cambodia: 0.30; w/w%, median, *p* < 0.001) (105). Moreover, the different dietary intakes of mothers living in different countries can significantly affect not only relative but also absolute TFA concentrations in HM, as demonstrated in an Asian study (C18:1 *t*: China: 84.0 ± 468.3, South Korea: 5.9 ± 21.6; Pakistan: 433.6 ± 438.5, Vietnam: 31.4 ± 44.0; mg/l, mean ± SD) (106).

The exclusive source of TFAs in HM is the dietary TFA intake, as discussed previously, therefore, dietary intake of both rTFAs and iTFAs can affect the total TFA content of HM. Mothers who follow a vegan diet, excluding meat, eggs, and dairy products (including milk) have significantly lower levels of TFAs in their HM samples compared to those who follow a vegetarian diet (including dairy products and eggs) or an omnivorous diet (52). There was a positive correlation between higher consumption of eggs or meat and higher levels of TFAs in HM (93). In contrast, diets high in grains, cereals, vegetables, legumes, nuts, and seeds resulted in a low TFA intake, and therefore, in lower TFA values in HM. Not only VA but EA and total TFA were directly associated with maternal milk and dairy intake (107). In Croatian mothers, the consumption of bakery, confectioners, fried foods, dairy products, margarine, and sausages was significantly directly related to the TFA content of HM (38). In Polish mothers, only a high intake of bakery products and snacks (rich in iTFAs, as EA) increased HM total TFA content significantly (67). High dairy fat intake (rich in rTFAs, as VA), on the other hand, can lead to a significant increase in VA content in HM (86). However, in Malaysian mothers with low TFA intakes (1.27 g/day), neither a healthy dietary pattern rich in vegetables and fruits nor frequent consumption of unhealthy deep-fried foods or eating out affected the EA and total TFA content of HM (108). In Brazilian adolescents with somewhat higher daily TFA intake (3.07 g/day), also no correlation was found between dietary intake and HM TFA contents (40). In an experimental study, a high-TFA diet given to lactating women significantly increased C18:1 *t* in HM samples collected as early as day two compared to the control group, demonstrating that dietary TFA is excreted into HM very rapidly, within 24 h of consumption (109).

It is a well-known fact that fat content, and the concentration of FAs in HM varies over time, depending on the lactation stage and several factors can influence its values, like gestational age at delivery, maternal diet and time of sampling (46, 110). Not only the physiologically important PUFAs, but TFAs can also change depending on the lactation stage (Table 3). The total TFA values in the literature range from 0.19 [mean w/w% (92)] to 3.81 [mean w/w% (95)] in C, with the lowest value in mothers with a traditional Chinese diet and highest value in German mothers with Western-type diet. Previous studies have reported varying results regarding the levels of TFAs in HM during lactation. Some studies have shown a significant decrease in TFA levels over time, with the highest values observed in the C (40, 111, 112, 114, 115), while others have reported a similar trend, but without statistical significance (44, 91). In contrast, some research groups have found opposite results, with the lowest TFA values measured in C increasing over time (67, 92, 116), or showing an initial decrease followed by a non-significant increase (113). Interestingly, in a Spanish study, the highest C18:1 *t* values were only found in very-preterm and preterm HM that was significantly decreasing over lactation, but they failed to show this trend in the term group (117). A decrease in TFA content may also be associated with the duration of lactation, as the maternal diet becomes more balanced and diverse throughout breastfeeding (107). Since there is no clear change among TFA levels in C, TM, and MM, this raises the possibility of other factors that could also influence TFA levels in HM (47, 89).

**Table 3:** Change in the values of *trans* isomers in human milk over lactation.

First author, Year	Place of study	Subgroups	Nr of mothers	Lactation stage	C18:1n9*t* (EA)	C18:1n7*t* (VA)	C18:2*tt* (linoelaidic acid)	Total TFA
Bousset-Alferes et al., 2022* (111)	Mexico	—	*n* = 33	C (1-5d)	1.334 (1.699)	—	0.196 (0.182)	1.529 (1.648)
				TM (5-15d)	0.503 (0.846)	—	0.246 (0.232)	0.748 (1.033)
				MM (>15d)	0.585 (1.042)	—	0.361 (0.535)	0.945 (1.368)
Chen et al., 1997* (92)	China	Hong Kong	*n* = 51	C (1-3d)	n.d.	n.d.	0.15 (0.012)	0.81 (0.30)
				TM (2w)	n.d.	n.d.	0.20 (0.16)	0.91 (0.79)
				MM (4w)	n.d.	n.d.	0.20 (0.16)	0.89 (0.67)
				MM (6w)	n.d.	n.d.	0.21 (0.12)	0.97 (0.49)
		Chongxing (Si Chuan)	*n* = 33	C (1-3d)	n.d.	n.d.	0.01 (0.02)	0.19 (0.25)
				TM (2w)	n.d.	n.d.	0.02 (0.02)	0.21 (0.04)
				MM (4w)	n.d.	n.d.	0.02 (0.03)	0.22 (0.04)
				MM (6w)	n.d.	n.d.	0.02 (0.03)	0.26 (0.05)
De Souza Santos da Costa et al., 2016* (40)	Brazil	—	*n* = 54	C (3d)	0.39 (0.08)	0.76 (0.08)	0.11 (0.04)	2.16 (0.19)
				MM (3 m)	0.26 (0.06)	0.67 (0.05)	0.10 (0.02)	1.65 (0.013)
Mihályi et al., 2015^#^ (112)	Pécs, Hungary	—	*n* = 87	C (1d)	0.55 (0.40)	n.d.	0.06 (0.03)	1.18 (0.51)
			*n* = 61	MM (6w)	0.51 (0.48)	n.d.	0.06 (0.03)	1.04 (0.47)
Minda et al., 2004^#^ (113)	Pécs, Hungary	—	*n* = 18	C (1d)	n.d.	n.d.	n.d.	1.69 (1.48)
				TM (14d)	n.d.	n.d.	n.d.	1.50 (0.65)
				MM (28d)	n.d.	n.d.	n.d.	2.06 (1.27)
Mojska et al., 2003^&^ (67)	Poland	Spring	*n* = 50	C (3-4d)	n.d.	n.d.	n.d.	1.37 (1.00–2.00)
			*n* = 38	MM (5-6w)	n.d.	n.d.	n.d.	2.59 (1.49–3.34)
			*n* = 34	MM (9-10w)	n.d.	n.d.	n.d.	2.36 (1.55–3.92)
		Autumn	*n* = 50	C (3-4d)	n.d.	n.d.	n.d.	1.80 (1.42–2.48)
			*n* = 40	MM (5-6w)	n.d.	n.d.	n.d.	2.41 (1.79–4.31)
			*n* = 35	MM (9-10w)	n.d.	n.d.	n.d.	2.77 (1.53–4.18)
Tinoco et al., 2008* (44)	Brazil	—	*n* = 37	C (1-5d)	n.d.	n.d.	n.d.	2.34 (0.75)
				MM (35-42d)	n.d.	n.d.	n.d.	2.19 (0.47)
Urwin et al., 2013^&^ (114)	China	River/lake	*n* = 42	C (3-5d)	3.98 (3.77–4.15)	n.d.	n.d.	n.d.
				TM (14d)	3.54 (3.27–3.66)	n.d.	n.d.	n.d.
				MM (25d)	3.37 (3.29–3.69)	n.d.	n.d.	n.d.
		Coastal	*n* = 42	C (3-5d)	3.50 (3.23–3.69)	n.d.	n.d.	n.d.
				TM (14d)	2.99 (2.53–3.56)	n.d.	n.d.	n.d.
				MM (28d)	2.80 (2.47–3.74)	n.d.	n.d.	n.d.
		Inland	*n* = 41	C (3-5d)	3.27 (3.03–3.57)	n.d.	n.d.	n.d.
				TM (14d)	2.96 (2.60–3.24)	n.d.	n.d.	n.d.
				MM (28d)	2.87 (2.56–3.14)	n.d.	n.d.	n.d.

*mean (SD), ^#^median (IQR), ^&^median (27–75 percentile); C, colostrum; d, day; EA, elaidic acid; m, month; MM, mature milk; n.d., no data; TFA, *trans* fatty acid; TM, transitional milk; VA, vaccenic acid; w, week.

Due to the different nutritional needs and metabolic capacities of preterm and term infants, the TFA content in HM may differ. It is essential that preterm infants receive higher amounts of some LCPUFAs, specifically DHA, in order to grow and develop at their optimal level (118). Consequently, preterm HM contains a higher concentration of DHA and other n-3 fatty acids compared to term HM (119–121) although other studies show no difference (43, 122, 123). However, preterm HM, may also contain higher levels of TFAs than term HM, which could potentially interfere with the metabolism and absorption of n-3 fatty acid (46). Although there have been few studies investigating the differences between TFA levels in preterm and term milk, the results are inconclusive. Some papers report no difference between the two groups (122, 124), while others have found higher levels in C in preterm infants (119), or even lower values during the first month of lactation (117) or only in MM (123). A systematic review summarizing EA values in preterm and term milk during lactation found lower values in the preterm group (preterm – C: 0.39 ± 0.02, TM: 0.26 ± 0.03; MM: 0.35 ± 0.04 vs. term – C: 1.95 ± 0.02, TM: 1.13 ± 0.02, MM: 1.16 ± 0.27; weighted least squares mean ± SEM), however, it should be noted that preterm data were based on a much smaller group of studies and included participants, so further studies are needed to demonstrate whether there is a difference between these two groups (125).

### 3.2 Relationship between *trans* isomers and polyunsaturated fatty acids in human milk

LA and ALA cannot be synthesized in the human body, and therefore, they are called EFAs. They can be further metabolized into their longer-chain metabolites, where AA is formed from LA, while EPA, and DHA are formed from ALA (Figure 2) (126). This metabolic process takes place within the hepatic endoplasmic reticulum through the action of desaturase and elongase enzymes, with LA and ALA competing for these enzymes (127). Although humans can convert ALA to its longer-chain metabolites (including EPA and DHA), this conversion efficiency is rather limited, being highest in the perinatal period and decreasing to about 1% by adulthood (128). Due to this fact, DHA in the maternal diet provides a much more efficient source of DHA for the developing fetal and neonatal neural tissue than equivalent amounts of ALA (129).

Animal studies have shown that TFAs can inhibit the activity of the Δ-6 desaturase enzyme, which is involved in the metabolism of PUFAs, and thus TFAs may affect the availability of longer-chain n-3 and n-6 metabolites. As n-3 and n-6 LCPUFAs play an important role in perinatal life, including the maturation of the nervous system and the development of visual acuity, TFAs may negatively affect this process. An indirect sign of this disturbed metabolism can be the relationship between TFA and LCPUFA values. Previous studies found inverse associations between TFAs and the most important n-3 and n-6 LCPUFAs in blood lipids of pregnant women during pregnancy (130) and in term newborns at delivery (130–132). This suggests that there may also be a correlation between TFAs and PUFAs in HM. Although only a relatively small number of studies have examined this relationship, most of them have found inverse associations between these FAs.

EA values correlated inversely to LA in C (*r* = −0.670, *p* < 0.001), and to AA in MM (*r* = −0.364, *p* < 0.05), while there was a strong positive correlation with ALA values in C (*r* = +0.844, *p* < 0.001) and TM samples (*r* = +0.844, *p* < 0.001), but not in MM. In contrast, DHA values were significantly inversely correlated to EA in C (*r* = −0.422, *p* < 0.05), but directly in TM (*r* = +0.615, *p* < 0.001) samples, while in MM no significant correlation was found (111). An early study found an inverse association between C18:1 *t* values and both LA (*r* = −0.29, *p* < 0.0001) and ALA values (*r* = −0.25, *p* < 0.001), but not with their longer chain metabolites, AA, EPA, or DHA in Canadian mothers (89). In contrast, a decade later, no association was found between C18:1 *t* and EFAs (LA, ALA) in American HM (90). However, studies investigating European lactating women found significant negative correlations between C18:1 *t* and LA, AA, ALA, and EPA values at the 6th week (63), 3rd month (38) and 6th month of lactation (133), but DHA values were only inversely correlated to C18:1 *t* values at the 6th week (63), 3rd month of lactation (38).

Some studies also investigated the relationship between the total TFA content of HM samples and the PUFA values. In C, significant negative correlation was found between total TFA content on the one hand and essential n-6 LA values on the other hand (44, 111), while no clear relationship was found with the other n-3 and n-6 PUFA values (Table 4). One study found no significant correlations with AA, ALA, EPA, and DHA values (44), while the other study found significant positive correlations were found with AA, ALA, n-6 LCPUFA and n-3 PUFA values and negative correlations with DHA (111). In contrast, only one study investigated the relationship in TM (111) and found a significantly positive relationship between on the one hand TFA and on the other hand ALA, and DHA values. MM showed the clearest correlations: total TFA was significantly negatively correlated with LA in all studies (9, 38, 44, 63, 111, 133), while AA (38, 63, 111, 133), ALA (9, 38, 63, 133), EPA (38, 63), and DHA (38, 63) were significantly negatively correlated with TFA values in most studies, and only one study found a significant positive correlation with ALA, EPA, and DHA (111). In contrast, in American mothers no correlations were found between total or individual TFA values and the LA, ALA, n-3, and n-6 PUFA values (90).

**Table 4:** Relationship between total TFA content and polyunsaturated fatty acids in human milk in different timepoints presented as correlation coefficients.

	C		TM	MM				
Fatty acids	Tinoco et al., 2008 (44)	Bousset-Alferes et al., 2022 (111)	Bousset-Alferes et al., 2022 (111)	Szabó É et al., 2007 (63)	Tinoco et al., 2008 (44)	Szabó et al., 2010 (133)	Krešic et al., 2013 (38)	Bousset-Alferes et al., 2022 (111)
LA (C18:2n-6)	−0.49*	−0.661***	−0.328	−0.34***	−0.35*	−0.230***	−0.275*	−0.377*
GLA (C18:3n-6)	n.d.	+0.621***	−0.196	n.d.	n.d.	+0.288***	n.d.	+0.379*
DHGLA (C20:3n-6)	n.d.	n.d.	n.d.	−0.53***	n.d.	−0.198***	n.d.	n.d.
AA (C20:4n-6)	−0.04	+0.758***	−0.106	−0.59***	+0.28	−0.356***	−0.440*	−0.521**
ALA (C18:3n-3)	+0.04	+0.837***	+0.867***	−0.35***	−0.12	−0.155***	−0.213*	+0.583***
EPA (C20:5n-3)	−0.26	+0.204	−0.236	−0.42***	+0.04	−0.046	−0.065*	+0.468**
DPA (C22:5n-3)	n.d.	n.d.	n.d.	n.d.	n.d.	−0.106*	n.d.	n.d.
DHA (C22:6n-3)	+0.16	−0.382*	+0.693***	−0.50***	+0.20	−0.006	−0.032***	+0.161
n-6 PUFA	−0.45*	−0.271	−0.315	−0.39***	−0.34*	n.d.	−0.122*	−0.313
n-6 LCPUFA	n.d.	+0.734***	−0.203	−0.61***	n.d.	n.d.	n.d.	+0.340
n-3 PUFA	n.d.	+0.735***	+0.710***	n.d.	n.d.	n.d.	−0.264*	+0.628***

*p < 0.05, **p < 0.01, ***p < 0.001; AA, arachidonic acid; ALA, alpha-linolenic acid; C, colostrum; DHA, docosahexaenoic acid; DHGLA, dihomo-gamma-linolenic acid; DPA, docosapentaenoic acid; EPA, eicosapentaenoic acid; GLA, gamma-linolenic acid; LA, linoleic acid; LCPUFA, long-chain polyunsaturated fatty acid; MM, mature milk; n.d., no published data; PUFA, polyunsaturated fatty acid; TM, transitional milk.

Thus, the results so far suggest that TFAs may interfere with the metabolism of n-3 and n-6 PUFAs in HM during lactation, but the paucity of studies and conflicting results for some FAs suggest that further large-scale studies are needed to establish the actual relationship.

## 4 Relationship between human milk TFA content and infant health

The TFA content of HM can be a major concern, as newborns and infants are particularly vulnerable to the health effects associated with high intakes (134). The adverse effect of iTFAs in the perinatal period is mainly related to the disrupted metabolism of LCPUFAs and thus to the lower availability of these FAs crucial for the nervous system and somatic development (10). Human studies have demonstrated a negative correlation between TFA consumption and LCPUFA levels in both full-term infants (135) and healthy children aged 1–5 years (136). Additionally, an inverse association has been observed between infant plasma TFA concentrations and birth weight (137) indicating a possible early growth impairment linked to TFAs. There is a significant direct association between the proportion of TFAs in HM and their presence in plasma TGs and PLs of breast-fed infants (9). In this regard, the TFA content in HM should be reduced by modifying the diet of breastfeeding mothers, as dietary FAs are the major determinant of the composition of lipids in HM under sufficient energy supply (95). Since the largest part of the total TFA content of HM is EA, the total TFA content is closely related to its iTFA content.

**Figure 4:**
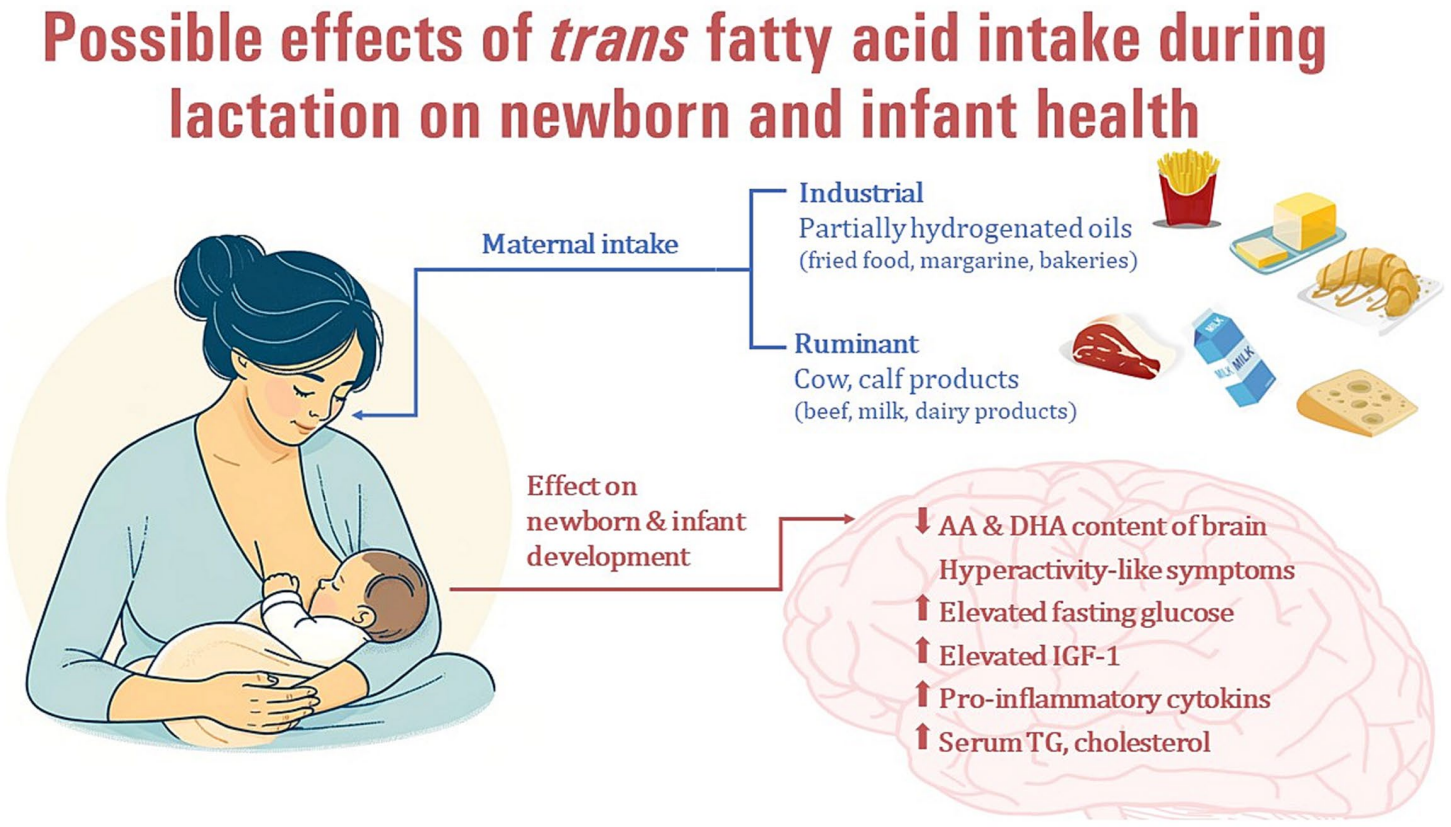
Possible effects of maternal industrial *trans* fatty acid intake during lactation on newborn and infant health.

During the prenatal stage, the fetus is exposed to TFAs through placental transfer, with the exposure relying on the concentration of the FAs in the mother’s bloodstream, which, in turn, is influenced by her dietary intake (132, 138, 139). TFA levels in the umbilical cord blood at birth reflect the intrauterine supply and the dietary TFA intake of the mother during pregnancy. Previous studies have found a positive association between maternal and neonatal blood TFA levels (132, 138), supporting a direct effect of maternal nutrition, including TFA intake, during pregnancy on neonatal FA supply. TFAs are able to cross the placenta and thus appear in the umbilical cord plasma (131, 132, 138, 140, 141) and red blood cell (RBC) samples at birth (130, 132) and are also incorporated into the umbilical cord wall (135, 142). Higher maternal TFA intake during pregnancy may therefore interfere with the LCPUFA supply in the newborn infant at birth, an indirect sign of which may be the negative correlation between TFAs and the levels of LCPUFAs (AA, EPA, and DHA) important for the development of the nervous system (130–132, 135, 137, 138). Similarly, TFA intake during breastfeeding through HM may affect the n-3 and n-6 LCPUFA supply of breastfed infants and thus may have a longer-term adverse effect on development. Some previous studies have reported possible negative effects of TFA levels during pregnancy or delivery on gestational age (141), placental weight (143), fetal head circumference (144) or birth weight (142, 143), raising the question of whether breast milk TFA levels also affect infants’ somatomental development (Figure 4).

### 4.1 Animal studies

Ethical considerations have played a decisive role in the preference for animal studies over human studies when investigating the effects of TFAs in milk on infant health. The use of animal models, such as rats, mice or pigs allows researchers to investigate the effects of TFAs on infant health without exposing human infants to potential risks.

Animal models can also be used to study the effects of dietary TFA intake during pregnancy or lactation and its incorporation into maternal tissues such as the MG. In a rat experiment, high TFA intake during the early stages of pregnancy (until day 12), resulted in TFAs accumulating in maternal adipose tissue and MG and mobilizing from these stores despite a subsequent (after day 12) TFA-free diet and appearing in maternal, fetal and neonatal plasma samples. Moreover, these previously stored TFAs were distributed to the MG, making them available to the suckling offspring (145).

Dietary TFA intake during lactation is mainly accumulated in maternal liver tissues and MG and is also excreted in milk, where it limits the availability of EFAs to suckling offspring. Maternal TFA supply mainly increased MG lipoprotein lipase activity in a dose-dependent manner, with a less pronounced effect in the liver and no effect on adipose tissue suggesting that these two factors (TFA storage and increased lipoprotein lipase activity in the MG due to TFA) may underlie the higher TFA content in milk samples (146). A study conducted on animals reported that a diet rich in TFAs during lactation and after weaning had an impact on the FA supply of newborn piglets. At 3 days of age, their plasma levels of AA and docosapentaenoic acid (C22:5n-3, DPA) were significantly lower. Furthermore, the consumption of a TFA diet affected the composition of aortic PL FAs. At 3 days of age, it increased LA values and at 48 days of age, it increased C18:1 *t* and LA values, but decreased AA, EPA, and DHA values (39). Therefore, high maternal TFA intake during lactation can not only influence the plasma fatty acid concentration of their newborns, but also incorporate TFAs into their cells, potentially disrupting the metabolism of EFAs into their longer chain metabolites, AA, EPA, and DHA.

#### 4.1.1 Brain development and behavior

It appears that higher maternal TFA intake during pregnancy and lactation may have a significant effect on the FA composition of the brain of the offspring. In an experimental study in which rats were fed a high-TFA diet or a control diet during pregnancy and lactation, the brains of 21-day-old offspring contained detectable amounts of TFAs. Moreover, high maternal TFA intake interfered with the incorporation of n-3 and n-6 LCPUFAs into the brain, leading to significantly lower AA and DHA levels in brain samples from the TFA group. However, when dams fed a high-TFA diet during pregnancy were switched to a normal (TFA-free) diet during lactation, EA levels in their adult offspring decreased, but AA and DPA levels increased significantly compared to the offspring of dams fed a high-TFA diet during both pregnancy and lactation. In fact, there was also a trend toward increased levels of EPA and DHA in this group, raising the possibility that the adverse effects of high TFA intake during pregnancy may be reduced by an adequate, low-TFA diet during lactation (147). This result was confirmed by another research group, where dietary TFA given to dams during lactation resulted in higher brain TFA levels in their adult offspring than in those who received a TFA diet only during the fetal period (during pregnancy). Moreover, the effect of TFA given only during lactation resulted in much lower AA and DHA content in the brains of adult offspring compared to the group that received TFA only during pregnancy (148). Similarly, a maternal TFA-rich diet during pregnancy and lactation increased TFA and decreased LA and ALA content in the offspring’s brain at 21 days of life (after weaning) and LA and EPA content in adulthood (149) suggesting that maternal diet during pregnancy and lactation through HM may have a lasting effect on the FA composition of the infants’ and children brain. Furthermore, the consumption of TFAs by mothers during pregnancy and lactation in two consecutive generations resulted in increased locomotor activity, impulsiveness, and agitation behavior in their offspring This suggests a correlation between TFA intake and hyperactivity-like symptoms (150). The FA composition of maternal diet during pregnancy and lactation, as well as the timing of brain development, can affect the incorporation of FAs into brain neural membranes. This can initiate early stages of inflammatory pathways in the brains of their offspring, ultimately impairing both short and long-term memory function (148, 149), increasing anxiety-like symptoms (151), and facilitating the development of hyperactivity-like symptoms (150) (Figure 4).

#### 4.1.2 Glucose metabolism, insulin sensitivity

Animal studies have also focused on the impact of TFAs in breast milk on offspring’s glucose metabolism and insulin parameters, providing valuable insights into their potential effects on early-life metabolic health. Maternal exposure to TFAs during lactation resulted in elevated fasting glucose levels in their offspring at weaning. However, HbA1c and insulin sensitivity remained unchanged, indicating a TFA-induced hepatic insulin resistance with adequate peripheral insulin sensitivity (152). In contrast, TFA intake via breastmilk did not affect serum glucose or insulin levels in rat pups, but insulin receptor and insulin receptor substrate-1 protein levels were significantly reduced in these rats (147). Additionally, adult offspring rats did not show any changes in blood glucose and insulin levels due to TFA intake during lactation (153). A study found that when mothers consumed TFAs during lactation, their adult offspring had significantly reduced levels of cardiac glucose transporter-4 levels, decreased hepatic glycogen content, and impaired insulin sensitivity. These results support the hypothesis, that early-life exposure to TFA-rich diets can lead to cardiac insulin resistance and have adverse consequences later in life (154).

Elevated plasma insulin-like growth factor-1 (IGF-1) in mouse pups with a high maternal TFA diet during lactation seemed to contribute to lower body fat, but this difference disappeared by adulthood (152). In pups of dams on a TFA diet during both pregnancy and lactation, there was no effect of this diet on appetite regulation, but when the newborn pups were switched from an intrauterine TFA diet to a postnatal control (TFA-free) diet, insulin-induced hypophagia disappeared, indicating that early TFA exposure may have long-term consequences on appetite regulation (147). In adult male rats on pre-and postnatal TFA-diet (including pregnancy and lactation) intracerebroventricular injection of insulin did not decrease 24-h feeding, although there was no difference in body weight compared to the control group (155). These studies underscore that early perinatal consumption of TFAs can affect food intake regulation in response to centrally administered insulin in young adult offspring, while the precise underlying mechanisms require further exploration, highlighting potential programming effects of early-life exposure to TFAs on hypothalamic feeding control mechanisms, which could result in adverse outcomes later in life.

#### 4.1.3 Body weight, adiposity

High maternal TFA intake during lactation delayed body weight gain only during the first week of life, but no difference was found later, although male offspring tended to be slightly smaller in adulthood (*p* = 0.06) compared to the group not receiving TFA during lactation. TFA-diet during lactation also affected the body composition in the offspring both at weaning and in adulthood, as measured by less abdominal fat (152). By contrast, in a rat study, maternal TFA-diet during pregnancy significantly decreased birth weight compared to n-6 PUFA of saturated fatty acid-rich diet, but at 21 days of age, these pups had significantly higher body weight, body weight gain, and relative weight of retroperitoneal fat compared to the n-6 PUFA group (156). However, others found no effect of perinatal TFA-intake on body weight in suckled pups (145, 153, 157) or adult offspring (147, 153, 155).

Maternal TFA-diet during pregnancy and lactation did not affect the weight of inguinal, retroperitoneal, epididymal, or mesenteric fat pads in their three-month-old male offspring. However, the mean areas of epididymal and inguinal adipocytes increased by 1.5 and 1.8-fold respectively, compared to the control diet-fed group. This suggests that maternal TFA-intake can enhance the deposition of visceral fat in their offspring, and therefore, may play a role in the development of later obesity (157).

#### 4.1.4 Blood lipids

The most well-known adverse effect of iTFAs is the increased risk of cardiovascular morbidity and mortality, which is due, among other things, to the increased levels of atherogenic blood lipids. Animal studies may also help to investigate the effects of a high maternal TFA diet during lactation on the blood lipids of their offspring in adolescence or even adulthood. Only prenatal maternal TFA diet in mice pups increased the circulating free fatty acid (FFA) concentration, but this difference disappeared by adulthood. In mice pups exposed to TFAs only during lactation, however, no significant alteration was measured (152). After weaning, in 48-day-old piglets, no difference in total cholesterol and high-density lipoprotein (HDL) cholesterol values were found between the TFA and control groups (39). By contrast, TFA-diet during pregnancy and lactation resulted in significantly higher TG, cholesterol and lower HDL cholesterol levels in 21-day old rat pups. Similarly, pre-, and postnatal TFA diets significantly increased FFA and cholesterol levels, while TG values were decreased in adult offspring. A postnatal TFA-free diet could decrease FFA and cholesterol values, but they remained non-significantly higher compared to controls (153). Although there are animal studies about the effect of maternal TFA intake on the blood lipids of their offspring at different ages, most studies supplemented dams during both pregnancy and lactation, so we cannot conclude that they show the direct effect of TFA in breast milk. Another disturbing effect is that many studies continue the same diet in the pups even after weaning, so it also cannot show a direct effect of early postnatal TFA intake via breast milk.

#### 4.1.5 Inflammation

Several studies have investigated the impact of maternal TFA consumption during pregnancy or lactation on the inflammatory status of offspring. In rat pups, maternal TFA intake during pregnancy and lactation increased the expression of pro-inflammatory adipokines such as plasminogen activator inhibitor-1 (PAI-1) and tumor necrosis factor-alpha (TNF-α) in white adipose tissue, but decreased the values and expression of anti-inflammatory adipokines, such as adiponectin or leptin (158). The changes observed were comparable to the low-grade inflammation status observed in obesity and metabolic syndrome. Therefore, it is possible that dietary TFA during the perinatal period may contribute to the perinatal programming of metabolic diseases. Similarly, maternal TFA diet in the pre-and postnatal period in 21-day-old rat offspring increased the expression of toll-like receptor 4 (TLR4), IL10Rα, and phosphorylated IκB kinase (p-IKKα+β) in the liver, although, there were no differences in the hepatic protein content of TNF-α, IL-6, and IL-10 (156). Based on a rat supplementation study, the long-term effects of pre-and postnatal exposure to TFAs might be different. Maternal TFA-supplementation during pregnancy or lactation significantly increased the pro-inflammatory cytokine levels (IL-1, IL-6, INF-γ, TNF-α) in both groups of adult offspring compared to controls, and this effect was more pronounced in the postnatal (TFA during suckling) group. By contrast, the anti-inflammatory IL-10 was decreased in both TFA groups, with significantly lower levels in the postnatal supplementation group. Moreover, increased reactive species generation, protein carbonyl levels, and catalase activity in the TFA-fed group also suggested increased oxidative stress in the adult offspring, that was even more pronounced when TFAs were consumed in the postnatal period via breastmilk (151).

The maternal TFA-diet during pregnancy and lactation may also impact the inflammatory state in the brain of the adult offspring. In an experimental study, the group fed with TFAs showed an increase in leucocyte rolling on the walls of brain venules, as well as an increase in leucocyte adhesion. These findings suggest leucocyte involvement in inflammation in the rat pups at 21 days of age (149). TFAs in the maternal diet during pregnancy and lactation may increase pro-inflammatory cytokine levels and decrease anti-inflammatory cytokine levels in the young and adult offspring. This may result in low-grade inflammation, but further research is needed to confirm these findings.

### 4.2 Human studies

Due to ethical considerations, human studies, contrary to animal studies, are not supplementation studies, but rather determine maternal TFA intake and/or HM TFA content and investigate their effects in early or late childhood. However, compared to animal studies, far fewer studies have examined these effects. As discussed above, the total TFA content of HM has been studied in several countries over lactation, in different ethnic groups, and in HM of preterm and term newborns. As previously stated, the largest portion of HM’s total TFA content is attributed to EA. Therefore, the adverse effects of HM’s total TFA content on infant development are primarily due to its iTFA content. Any potential beneficial effects of rTFA are discussed separately in the text.

Several studies have examined the relationship between maternal plasma TFA levels during pregnancy or at delivery and key pregnancy outcomes, including gestational age at birth, birth weight, and fetal head circumference. Higher maternal TFA levels in plasma may be associated with a shorter gestation period (138, 141), reduced birth weight, and small for gestational age at birth (159). A systematic review and meta-analysis (160) found three studies about the inverse correlation between maternal TFAs during pregnancy and birth weight, while one study did not find any association. Moreover, the cord erythrocyte C18:1 *t* value, which is a better indicator of the newborn’s supply, may also be inversely associated with birth weight (142). Higher maternal TFA levels during pregnancy may be related to lower fetal head circumference, but prenatal TFA exposure did not seem to affect global brain volume or nonverbal IQ in children (144). By contrast, neither cord plasma TFA (138) nor cord erythrocyte membrane C18:1 *t* (142) influenced head circumference at birth. From these studies, we can conclude that there might be a disturbing effect of maternal and neonatal TFA supply during pregnancy or at delivery on the infant development, but the effect of TFA intake through HM is a less researched topic and only a few studies can be found about short-and longer-term effects.

Maternal consumption of TFAs greater than 4.5 g/day during lactation appears to increase the odds ratio of higher percent body fat in both mothers (odds ratio: 5.81) and their 3-month-old infants (odds ratio: 2.13) for body fat percentages of 30% or greater in mothers and 24% or greater in infants (161). This study suggests that consuming TFAs during lactation may affect the body composition both mother and infant in the early postpartum period, potentially contributing to childhood and later obesity. However, no such relationship was found in Nigerian mothers regarding HM TFAs and maternal anthropometric values (97).

In a large birth cohort study (*n* = 1,369) maternal TFA intake during the second trimester was associated with higher fetal growth (162). In contrast, in a Norwegian birth cohort study (*n* = 789) no association was found between TFA content of HM samples and infant growth or rapid infant growth (163).

Although there were some correlations between maternal TFA supply during pregnancy and neonatal birth dimensions as discussed above, no relationship was found between TFAs in HM and head circumference, gestational age, and birth weight in term (40) as well as weight and height of preterm infants (44).

Animal studies, as discussed above, suggested an increased low-grade inflammation in those offspring that had higher TFA exposure during weaning (mainly iTFA, like EA), which might lead to an increased risk of developing allergies in infancy or childhood. However, rTFAs, like RA, and VA might be preventive. Increased concentration of both RA and VA in HM was correlated with a lower risk of eczema, atopic dermatitis, and allergic sensitization at one year of age, which was largely independent of HM n-3 LCPUFA values (164). Increased maternal VA-levels during pregnancy decreased the risk of atopic eczema in one-year-old children, but no other associations were found with other TFA values (165). Similarly, in children of mothers with allergy HM TFA content was associated with a significantly decreased odds ratio (OR: 0.57 95%CI: 0.33–1.00) of eczema at the age of four years, although no information is available, on whether these TFAs were iTFAs of rTFAs (C18:1 *t*, C16:1n-7 *t*, C18:2n-6 *t*) (166). By contrast, no relationship between maternal TFA intake during pregnancy and cow’s milk allergy in their three-years-old children was found (167).

## 5 Conclusion

Thanks to recent regulations, the general population, including breastfeeding women, is now exposed to very low levels of dietary iTFA in most parts of the world. The effectiveness of iTFA regulation in food can be tracked by monitoring the iTFA content of HM samples in the literature. While early articles reported higher values, more recent studies have indicated much lower values. Although the TFA content of HM may change during breastfeeding, the data on the direction of this change are conflicting. There are contradictory data on the relationship between TFAs and n-3 and n-6 PUFAs, but most articles report a negative correlation between iTFAs and LA, AA, and DHA. The effects of iTFA supplementation during lactation have only been investigated in animal studies due to ethical considerations. These studies have shown that higher iTFA intake through breastmilk can lead to negative effects in the offspring, including altered brain fatty acid content, anxiety-like symptoms, increased visceral fat deposition, higher blood triglyceride and cholesterol levels, and pro-inflammatory status. However, few human studies have investigated the effects of breast milk iTFA content on infant and toddler health. While some data suggest that maternal iTFA intake may interfere with infant fatty acid supply, body composition, and the development of allergic diseases, further studies are needed to explore this relationship.

## Author contributions

OH: Data curation, Writing – original draft. ÖFK: Data curation, Writing – original draft. HKK: Data curation, Writing – original draft. JLS: Visualization, Writing – review & editing. TM: Data curation, Software, Writing – review & editing. ÉS: Conceptualization, Data curation, Supervision, Visualization, Writing – review & editing.
