# Supplementary material for: Trans isomeric fatty acids in human milk and their role in infant health and development
Source: Front Nutr. 2024 Mar 7;11:1379772. doi: 10.3389/fnut.2024.1379772 (PMC10954868; doi:10.3389/fnut.2024.1379772)
Supplement: Supplementary file 1 [file Table_1.docx]

**Supplementary table 1**: Sum of *trans* fatty acids (total TFA) in human milk worldwide (presented as weight% of total fatty acids)

| **First author, Year** | **Place of study** | **Subgroup of mothers** | **Nr of mothers** | **Lactation stage** | **Total TFA** |
| --- | --- | --- | --- | --- | --- |
| Tinoco SMB et al, 2008*, (40) | Brazil |  | n = 37 | C (1-5d) | 2.34 (0.75) |
|  |  |  |  | MM (35-42d) | 2.19 (0.47) |
| De Souza Santos da Costa R et al,  2016*, (38) | Brazil |  | n = 54 | C (3d) | 2.16 (0.19) |
|  |  |  |  | MM (3m) | 1.65 (0.013) |
| Ratnayake WM and Chen ZY, 1996*, (77) | Canada |  | n = 198 | MM (3-4w) | 7.19 ± 3.03 |
| Ratnayake WM et al, 2014*, (71) | Canada | in 2009 | n = 153 | MM (2-8w) | 2.7 (0.9) |
|  |  | in 2010 | n = 309 |  | 2.2 (0.7) |
|  |  | in 2011 | n = 177 |  | 1.9 (0.5) |
| Chen ZY et al, 1997*, (80) | China, Hong Kong |  | n = 51 | C (1-3d) | 0.81 (0.30) |
|  |  |  |  | TM (2w) | 0.91 (0.79) |
|  |  |  |  | MM (4w) | 0.89 (0.67) |
|  |  |  |  | MM (6w) | 0.97 (0.49) |
|  | China, Chongqing |  | n = 33 | C (1-3d) | 0.19 (0.25) |
|  |  |  |  | TM (2w) | 0.21 (0.04) |
|  |  |  |  | MM (4w) | 0.22 (0.04) |
|  |  |  |  | MM (6w) | 0.26 (0.05) |
| Yip PSP et al, 2020*, (103) | China, Hong Kong |  | n = 60 | MM (4w) | 0.98 (0.31) |
|  |  |  |  | MM (6w) | 1.17 (0.36) |
| Krešić G et al, 2013*, (36) | Croatia |  | n = 83 | MM (3m) | 2.3 (0.2) |
| Marhol P et al, 2007*, (87) | Czech Republic | Rome women | n = 21 | C / TM (3-10d) | 3.78 (1.88) |
|  |  | Czech women | n = 43 |  | 3.13 (1.26) |
| Koletzko B et al, 1988^&^, (82) | Germany, Düsseldorf |  | n = 15 | MM (3-4m) | 4.40 (3.16-5.11) |
| Precht D & Molkentin J, 1999*, (83) | Germany, Berlin |  | n = 40 | C (1w) | 3.81 (0.97) |
| Szabo É et al, 2007^§^, (60) | Germany, Ulm | Others | n = 73 | MM (6w) | 1.79 (0.49-5.40) |
|  |  | German, born elsewhere | n = 65 |  | 1.74 (0.52-6.52) |
|  |  | German, born in Germany | n = 602 |  | 1.55 (0.34-5.89) |
|  |  | Turkish | n = 29 |  | 1.30 (0.42-5.44) |
| Szabó É et al, 2010^#^, (117) | Germany, Ulm |  | n = 462 | MM (6m) | 1.43 (1.55) |
| Antonakou A et al, 2013*, (79) | Greece, Athens |  | n = 64 | MM (1m) | 0.78 (0.47) |
|  |  |  | n = 39 | MM (3m) | 0.48 (0.50) |
|  |  |  | n = 24 | MM (6m) | 0.19 (0.34) |
| Minda H et al, 2004^#^, (104) | Hungary, Pécs |  | n = 18 | C (1d) | 1.69 (1.48) |
|  |  |  |  | C (2d) | 1.59 (1.22) |
|  |  |  |  | C (3d) | 1.57 (1.07) |
|  |  |  |  | C (4d) | 1.41 (0.91) |
|  |  |  |  | C (5d) | 1.70 (0.75) |
|  |  |  |  | C (6d) | 1.32 (0.87) |
|  |  |  |  | C (7d) | 1.38 (1.04) |
|  |  |  |  | TM (14d) | 1.50 (0.65) |
|  |  |  |  | MM (28d) | 2.06 (1.27) |
| Mihályi K et al, 2015^#^, (102) | Hungary, Pécs |  | n = 87 | C (1d) | 1.18 (0.51) |
|  |  |  | n = 61 | MM (6w) | 1.04 (0.47) |
|  |  |  | n = 46 | MM (6m) | 0.96 (0.40) |
| Bahrami G & Rahimiv Z, 2005*, (76) | Iran |  | n = 52 | MM (45-135d) | 11.3 (3.4) |
| Hayat L et al, 1999*, (86) | Kuwait |  | n = 19 | MM (6-14w) | 2.80 (1.75) |
| Aumeistere L et al, 2019^§^, (81) | Latvia |  | n = 61 | MM (>1m) | 0.20 (<0.10-1.50) |
| Aumeistere L et al, 2021^#^, (95) | Latvia |  | n=71 | MM (>1m) | 2.30 (0.60) |
| Daud AZ et al, 2013*, (96) | Malaysia, Selangor |  | n = 101 | MM (15d-6m) | 2.93 (0.96) |
| Bousset-Alferes CM et al, 2022*, (101) | Mexico |  | n = 33 | C (1-5d) | 1.529 (1.648) |
|  |  |  |  | TM (5-15d) | 0.748 (1.033) |
|  |  |  |  | MM (>15d) | 0.945 (1.368) |
| Glew H et al, 2006^@^, (85) | Nigeria, Jos | Urban (Jos) women | n = 41 | MM | 0.34 |
|  |  | Fulani (rural) women | n = 41 |  | 0.22 |
| Mojska H et al, 2003^&^, (64) | Poland | Spring | n = 50 | C (3-4d) | 1.37 (1.00–2.00) |
|  |  |  | n = 38 | MM (5-6w) | 2.59 (1.49–3.34) |
|  |  |  | n = 34 | MM (9-10w) | 2.36 (1.55–3.92) |
|  |  | Autumn | n = 50 | C (3-4d) | 1.80 (1.42–2.48) |
|  |  |  | n = 40 | MM (5-6w) | 2.41 (1.79–4.31) |
|  |  |  | n = 35 | MM (9-10w) | 2.77 (1.53–4.18) |
| Mueller A et al, 2010*, (74) | The Netherlands | Conventional diet | n = 185 | MM (1m) | 3.26 (1.06) |
|  |  | 0-10 g/d dairy fat intake | n = 55 |  | 3.27 (1.28) |
|  |  | 10-20 g/d dairy fat intake | n = 106 |  | 3.25 (0.98) |
|  |  | 20-40 g/d dairy fat intake | n = 117 |  | 3.11 (0.81) |
|  |  | 40-76 g/d dairy fat intake | n = 32 |  | 3.06 (0.63) |
|  |  | 50-90% Organic | n =33 |  | 3.05 (1.03) |
|  |  | > 90% Organic | n =37 |  | 3.14 (0.66) |
| Samur G et al, 2009*, (11) | Turkey |  | n = 50 | MM (12-16w) | 2.13 (1.03) |
| Mosley E et al, 2005*, (78) | USA |  | n = 81 | MM (1m) | 7.0 (2.3) |
| Perrin TM et al, 2019^#^, (48) | USA | Omnivore | n = 26 | MM (>2w) | 1.09 (0.55) |
|  |  | Vegetarian | n = 22 |  | 0.66 (0.71) |
|  |  | Vegan | n = 26 |  | 0.44 (0.19) |

*: mean (SD), ^@^: mean, ^#^: median (IQR), ^&^: median (27-75 percentile), ^§^: median (range)

C: colostrum, d: day, m: month, MM: mature milk, n.d.: no data, TFA: total *trans* fatty acids, TM: transitional milk, w: week
